# Determining the Prognostic Value of Spliceosome-Related Genes in Hepatocellular Carcinoma Patients

**DOI:** 10.3389/fmolb.2022.759792

**Published:** 2022-02-24

**Authors:** Jun Liu, Liming Gu, Dangui Zhang, Wenli Li

**Affiliations:** ^1^ Reproductive Medicine Center, Yue Bei People’s Hospital, Shantou University Medical College, Shaoguan, China; ^2^ Guangdong Provincial Key Laboratory of Infectious Diseases and Molecular Immunopathology, Shantou University Medical College, Shantou, China; ^3^ Medical Research Center, Yue Bei People’s Hospital, Shantou University Medical College, Shaoguan, China; ^4^ Department of Microbiology and Immunology, Center of Pathogen Biology and Immunology, Shantou University Medical College, Shantou, China; ^5^ Research Center of Translational Medicine, Second Affiliated Hospital of Shantou University Medical College, Shantou, China

**Keywords:** snRNP, prognostic genes, HCC, TMB, immune cell

## Abstract

**Background:** The spliceosome plays an important role in mRNA alternative splicing and is aberrantly expressed in several tumors. However, the potential roles of spliceosome-related genes in the progression of hepatocellular carcinoma (HCC) remain poorly understood.

**Materials and Methods:** Patient data were acquired from public databases. Expression differences and survival analyses were used to assess the importance of spliceosome-related genes in HCC prognosis. To explore the potential regulatory mechanisms of these genes, a protein-protein interaction network was constructed and screened using univariate and multivariate Cox regression and random forest analyses. This was used to create a five-gene prognostic model. The prognostic value and predictive power of the five-gene signature were assessed using the Kaplan-Meier and time-dependent receiver operating characteristic analyses in the training set. These results were further validated in an independent external set. To facilitate clinical application, a nomogram was prepared to predict the overall survival of HCC patients. The relative expression of five genes was detected using real-time quantitative polymerase chain reaction.

**Results:** The analysis revealed that *LSM1-7, SNRPB, SNRPD1-3, SNRPE, SNRPF, SNRPG,* and *SNRPN* could be used as prognostic biomarkers in HCC patients. Moreover, the five-gene risk model could clearly distinguish between the high-and low-risk groups. Furthermore, the risk model was associated with the tumor mutation burden, immune cell infiltration of CD8^+^ T cells, natural killer T cells, M2 macrophages, and immune checkpoint inhibitors, which also demonstrated the predictive efficacy of this risk model in HCC immunotherapy.

**Conclusion:** Spliceosome-related genes and the five-gene signature could serve as novel prognostic biomarkers for HCC patients, aiding clinical patient monitoring and follow-up.

## Introduction

Hepatocellular carcinoma (HCC) is a malignant tumor that is associated with high morbidity and low advanced survival rates worldwide (Bray et al., 2018). The lack of biomarkers for early diagnosis and prognosis presents a challenge for medical practitioners (Nault and Villanueva, 2015). Investigators have exhaustively researched reliable prognostic indicators that may help in determining the risk of adverse outcomes in patients using tissue or blood samples (O'Brien et al., 2019; Ayoub et al., 2019). Unfortunately, universally acceptable biomarkers or predictive models have not been found yet.

The spliceosome is an organelle-like complex that plays a significant role in regulating gene expression and producing protein diversity (Nilsen and Graveley, 2010). The spliceosome consists of five small nuclear ribonucleoproteins (snRNP; U1, U2, U4, U5, and U6), and the Lsm/sm proteins form the core scaffold of snRNP (Roth et al., 2018). These molecules, including the snRNPs and Lsm proteins, are involved in the splicing of pre-mRNA. SnRNP genes have been regarded as oncogenic in glioblastoma (Correa et al., 2016), and their high expression is also correlated with poor prognosis in non-small cell lung cancer (Valles et al., 2012). However, contradictory to these findings, snRNPs were identified as metastasis suppressor genes in prostate cancer (Yi et al., 2009), highlighting their diverse roles. To date, there have been few reports on the involvement of snRNPs in the progression of HCC and its clinical significance (Peng et al., 2020). In addition, the precise role of Lsm proteins in HCC has not yet been established.

In the current study, we determined the expression of snRNPs and Lsm proteins in HCC and its implication in the prognosis of HCC patients. We measured the association between the expression of snRNPs and Lsm proteins and various clinical characteristics of HCC patients. Further we determined the prognostic value of these markers by using survival curves and Cox regression analysis. We also analyzed the genes with expression patterns similar to those of snRNPs and Lsm proteins to find potential regulatory networks. We successfully extracted a five-gene signature from the co-expressed genes, which will aid clinicians in prognosis assessment.

## Materials and Methods

### Acquisition of Patient Data

The RNA-seq of HCC, single nucleotide mutation, and clinical data of patients were downloaded from The Cancer Genome Atlas (TCGA) (https://cancergenome.nih.gov). Normal tissues adjacent to the tumor were available for 50 patients, which were used as controls. Samples with insufficient information were excluded from the analysis. Expression and clinical data (ICGC-LIRI-JP) acquired from the International Cancer Genome Consortium (ICGC) (https://www.icgc.org) were used for validation.

### Analysis of Expression Difference

We used data from TCGA database to analyze the differences in expression of proteins between tumors and normal tissue. We verified these results using data from the ICGC database. Protein expression was compared using the Wilcoxon signed-rank test.

### Prognosis and Biological Function Analysis and Protein-Protein Interaction Network Construction

Comparison of overall survival (OS) of patients with high or low snRNPs and Lsm expression was conducted using the Kaplan-Meier (K-M) survival analysis. The best separation was used to define the high-and low-risk groups. The relationship between clinicopathologic features and snRNPs and Lsm was analyzed using the Kruskal test. Gene ontology (GO) was used to identify potential molecular functions of snRNPs and Lsm involved in HCC development. The GeneMANIA database (http://genemania.org) (Warde-Farley et al., 2010) was used to construct a protein-protein interaction network (PPI) and render significant pathways.

### Generation and Validation of the Gene Signature

Univariate Cox regression was used to screen the genes associated with OS from the PPI network in the TCGA cohort. Next, the relative importance of OS-related genes was identified and ranked based on the random forest algorithm. Subsequently, multivariate Cox regression analysis was conducted to construct a risk model: risk score = β1×1 + β2×2 + β3×3 +,…,+ βnxn. The HCC patients with survival data were separated into low- and high-risk groups based on the median risk score. The K-M survival and receiver operator characteristic (ROC) curves for the cases were obtained. The ICGC cohort was used as an external validation cohort in the analysis. Univariate and multivariate Cox regression analyses were used to compare the influence of the signature on survival along with other clinical characteristics.

### Correlation Analysis Between the Gene Signature and Tumor Mutation Burden and Immune Cell Infiltration

The tumor mutation burden (TMB) was calculated from single nucleotide mutations, and the distribution of TMB and somatic mutations between high- and low-risk groups was analyzed in the TCGA HCC cohort using the “maftools” R package. Simultaneously, single-sample gene set enrichment analysis (ssGSEA) and CIBERSORT algorithms were used to assess the extent of immune cell infiltration.

### Construction and Evaluation of a Predictive Nomogram

A combined model comprising all independent prognostic factors was constructed using the rms package to assess the probability of OS in HCC patients. Discrimination and calibration were performed to evaluate the nomograms. Discrimination of the nomogram was performed using decision curve analysis (DCA). The calibration curve of the nomogram was visualized by plotting the nomogram prediction probabilities against the observed rates.

### Cell Lines and Cell Culture

The normal human cell line LO2 and HCC cells, HCCLM3, Hep-G2, and Huh7 were purchased from the American Type Culture Collection (ATCC, Manassas, VA, United States). All the cells were cultured at 37°C under 5% CO2.

### Quantitative Reverse-Transcription Polymerase Chain Reaction

The PCR primers for *SNRPB, LSM10, ATXN2, PRPF3, EDC3*, and β action were designed with NCBI primer Blast tool. Detailed primer sequences of these genes were listed in Supplement Table S1. The total RNA was extracted from 4 cell lines, subsequently, RNAs were reverse transcribed into cDNA through reverse transcription Kit (Beyotime, https://www.beyotime.com/). Then the relative expression of these genes was detected using Real-Time Quantitative polymerase Chain Reaction (RT-qPCR).

## Results

### Patient Characteristics

We used data mining to analyze the prognostic significance of hepatic *SNRPB* in HCC patients. Data for 370 primary tumors including clinical and gene expression data were downloaded from the TCGA databank. Similarly, data on 232 patients were downloaded from the ICGC databank. The patients with TCGA cohort were draw from United States, and were predominantly Caucasian and African-American. While, the patients with ICGC cohort come from Japan, and were predominantly Asian. The clinical data for patients, including survival status, age, sex, histological grade, stage, and prior malignancy history, have been described in Table 1.

**TABLE 1:** The clinical baseline characteristics of HCC patients.

Clinical characteristics		TCGA	%	ICGC	%
		370		232	
Survival status	Survival	244	65.95	189	81.47
	Death	126	34.05	43	18.53
Age	≤65years	232	62.70	90	38.79
	>65 years	138	37.30	142	61.21
Gender	Male	249	67.30	171	73.71
	Female	121	32.70	61	26.29
Histological grade	G1	55	14.86	NA	
	G2	177	47.84	NA	
	G3	121	32.70	NA	
	G4	12	3.24	NA	
Stage	Ⅰ	171	46.22	36	15.52
	Ⅱ	85	22.97	106	15.69
	Ⅲ	85	22.97	71	30.60
	Ⅳ	5	1.35	19	8.19
Prior malignancy	No	NA	NA	202	87.07
	Yes	NA	NA	30	12.93

Abbreviations: TCGA, the cancer genome atlas; ICGC, international cancer genome consortium.

### Transcriptional Expression Landscape of Spliceosome Related Genes

**FIGURE 1:**
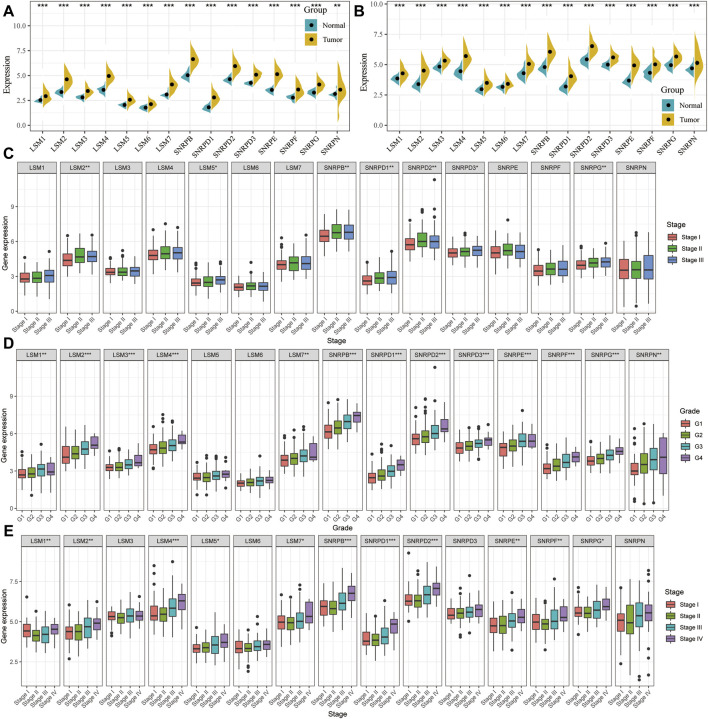
Comparison of spliceosome-related genes in the TCGA and ICGC cohort. Expression difference of LSM1-7, SNRPB, SNRPD1-3, SNRPE, SNRPF, SNRPG, and SNRPN between HCC tumor and non-tumor tissues in TCGA **(A)** and ICGC **(B)** cohort. tumor and paired tumor-adjacent tissues from the TCGA cohort. Comparison of spliceosome-related genes expression of patients with different clinical stages **(C)** and histologic grade **(D)** from the TCGA cohort. **(E)** Comparison of spliceosome-related genes expression of patients with different clinical stages from the ICGC cohort. **(G)** means histological grade, and stage means pathologic TNM staging. *p < 0.05, **p < 0.01, ***p < 0.001.

The expression levels of *LSM1-7, SNRPD1-3, SNRPB, SNRPF, SNRPG, SNRPE*, and *SNRPN* were compared between cancer and non-cancer liver tissues in HCC patients from the TCGA and ICGC cohorts. The mRNA expression of all 15 genes in the cancer tissues was significantly higher than that in the non-cancer tissues in both the TCGA and ICGC cohorts (Figures 1A,B). We then compared the expression of all genes at different tumor stages and grade classifications. The results indicated that the expression levels of *LSM2, LSM5, SNRPB, SNRPD1, SNRPD2, SNRPD3,* and *SNRPG* were higher in tumors at more advanced clinical stages in the TCGA cohort (Figure 1C). Further, increased expression of *LSM1, LSM2, LSM4, LSM5, LSM7, SNRPB, SNRPD1, SNRPD2, SNRPE, SNRPF,* and *SNRPG* significantly correlated with stage classification in the ICGC cohort (Figure 1E). We also found that high expression of *LSM1, LSM2, LSM4, LSM5, LSM7, SNRPB, SNRPD1, SNRPD2, SNRPE, SNRPF*, and *SNRPG* was prevalent in tumors with higher histologic grades (Figure 1D). Thus, we concluded that snRNPs and LSM were overexpressed in HCC tumors, and their expression was significantly correlated with clinical stage and grade classification.

### Prognostic Value of Spliceosome Related Genes

**FIGURE 2:**
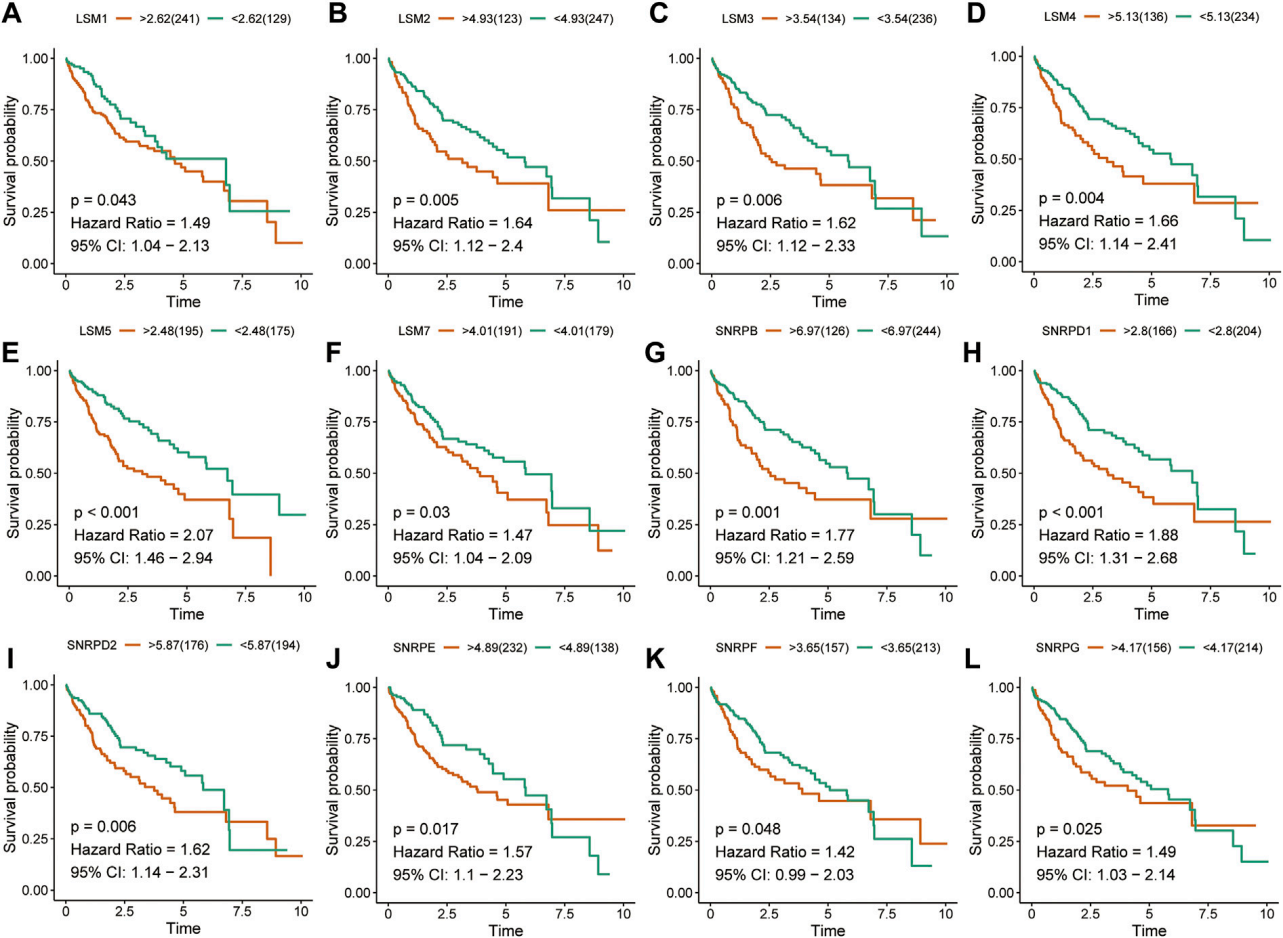
Survival analysis of spliceosome-related genes for HCC patients in TCGA cohort. Overall survival of LSM1 **(A)**, LSM2 **(B)**, LSM3 **(C)**, LSM4 **(D)**, LSM5 **(E)**, LSM7 **(F)**, SNRPB **(G)**, SNRPD1 **(H)**, SNRPD2 **(I)**, SNRPE **(J)**, SNRPF **(K)**, and SNRPG **(L)** were performed by Kaplan-Meier plotter.

**FIGURE 3:**
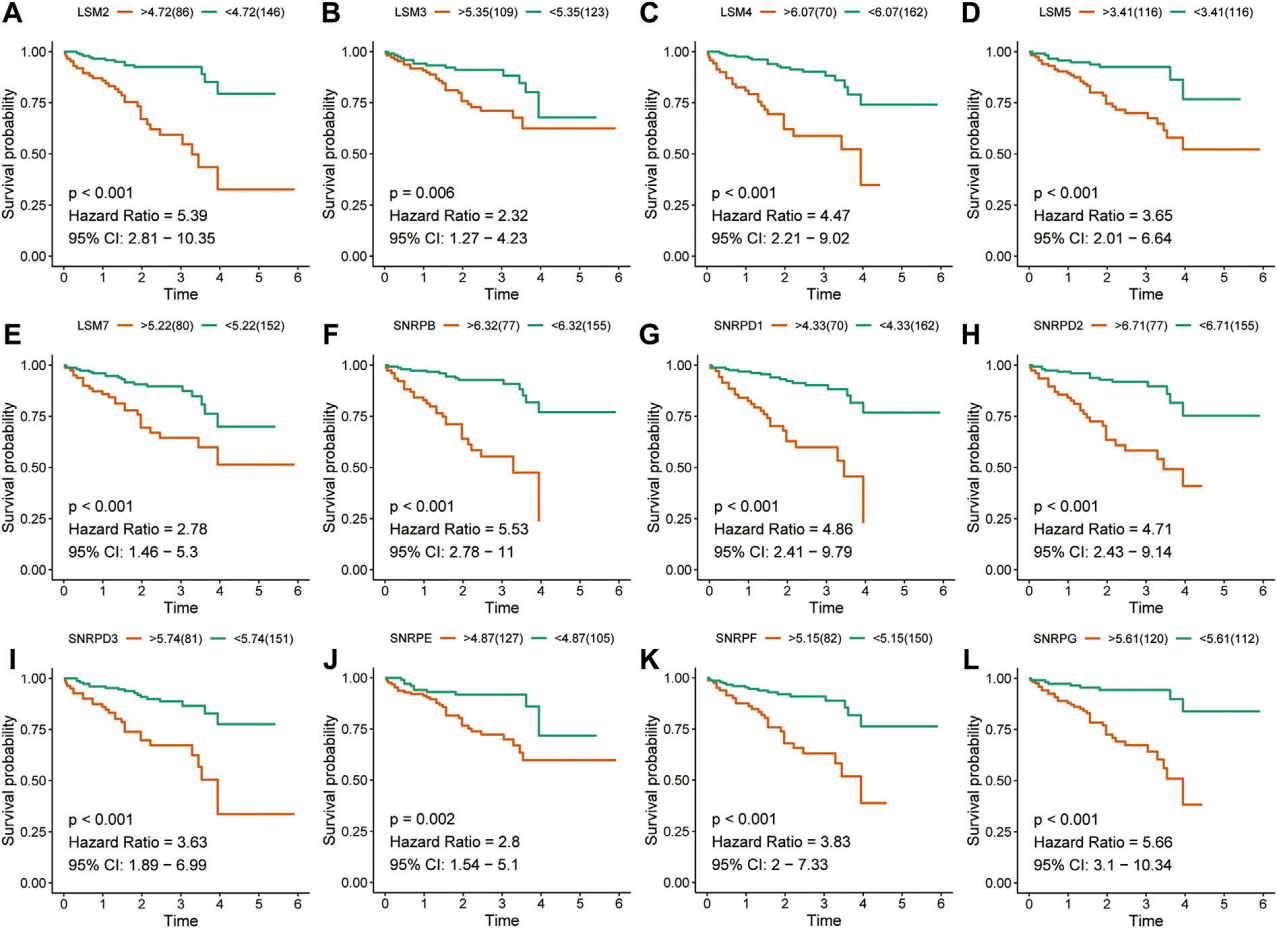
Survival analysis of spliceosome-related genes for HCC patients in ICGC cohort. Overall survival of LSM2 **(A)**, LSM3 **(B)**, LSM4 **(C)**, LSM5 **(D)**, LSM7 **(E)**, SNRPB **(F)**, SNRPD1 **(G)**, SNRPD2 **(H)**, SNRPD3 **(I)** SNRPE **(J)**, SNRPF **(K)**, and SNRPG **(L)** were performed by Kaplan-Meier plotter.

The K-M survival curves suggested that HCC patients with higher expression of *LSM1-5, LSM7, SNRPB, SNRPD1, SNRPD2, SNRPE, SNRPF*, and *SNRPG* showed shorter survival rates in the TCGA cohort (Figures 2A-L). Similar results were confirmed in the ICGC cohort: patients with increased *LSM2-5, LSM7, SNRPB, SNRPD1, SNRPD2, SNRPE, SNRPF*, and *SNRPG* expression had shorter OS (Figures 3A-L). In addition, high *SNRPD3* expression was associated with poor prognosis in the ICGC cohort (Figure 3I). These results further validated the prognostic value of *LSM2-5, LSM7, SNRPB, SNRPD1, SNRPD2, SNRPE, SNRPF,* and *SNRPG* in HCC.

### Co-Expression Correlation and Functional Enrichment Analysis

**FIGURE 4:**
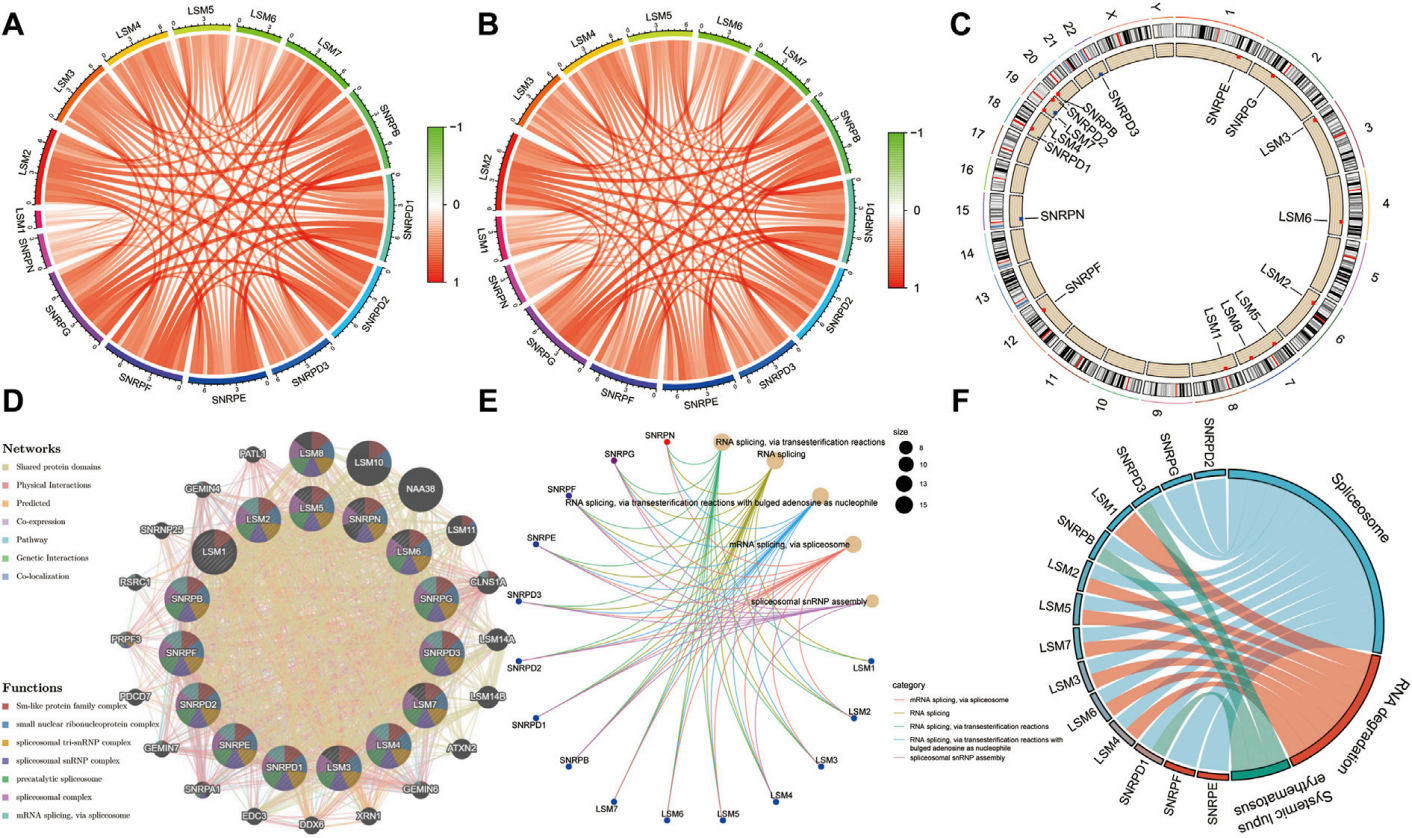
| Expression correlation among spliceosome-related genes and construction of protein-protein interaction (PPI) network. Expression correlation among spliceosome-related genes in the TCGA **(A)** and **(B)** cohort. **(C)** The location of spliceosome-related genes on chromosomes. **(D)** The PPI network of spliceosome-related genes was built based on the GeneMANIA database. **(E)** Gene ontology enrichment analysis of spliceosome-related genes. **(F)** KEGG enrichment analysis of spliceosome-related genes.

Co-expression analysis was performed to identify associations among these genes, and the results indicated that there was a very strong correlation among these subunit genes in both the TCGA and ICGC cohorts (Figures 4A,B). The locations of the 16 subunit genes on chromosomes are shown in Figure 4C. To further understand the role of these genes in the development of liver cancer, a protein-protein interaction network was constructed using the GeneMANIA database (Figure 4D). Functional enrichment analysis revealed that these molecules were mainly involved in mRNA splicing, the spliceosomal complex, and Sm-like protein family complex, as expected. GO analyses also showed that these genes were mainly associated with RNA splicing and spliceosomal snRNP assembly (Figure 4E). Meanwhile, KEGG analysis also demonstrated that these genes were involved in Spliceosome, RNA degradation, and Systemic lupus erythematosus (Figure 4F).

### Generation and Validation of a Prognostic Signature

**FIGURE 5:**
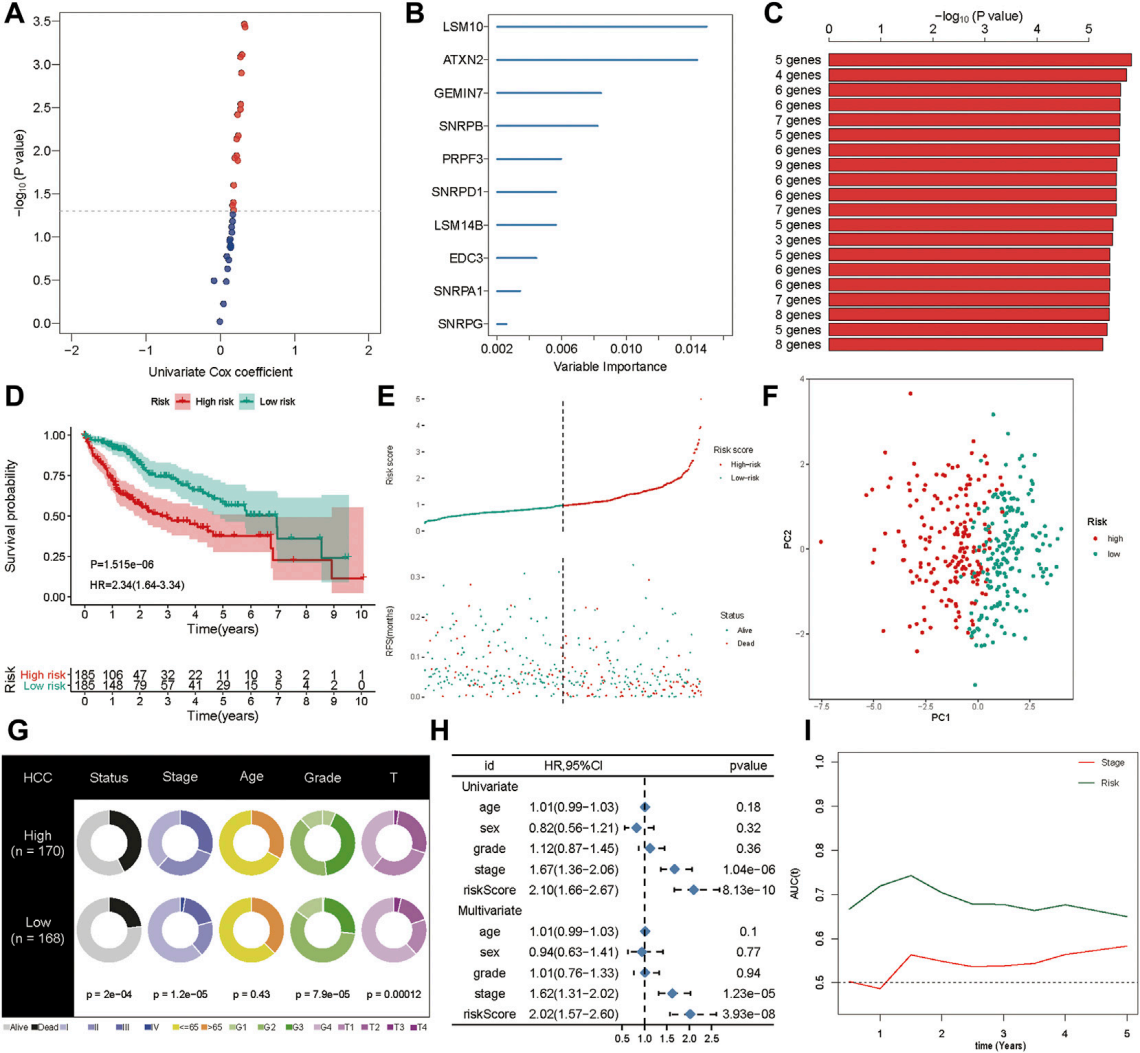
Generation of the prognostic signature. **(A)** The univariate Cox regression was applied to filter spliceosome-related genes related to overall survival. **(B)** The random forest was used to screen and rank the relative important gene for overall survival. **(C)** The Kaplan-Meier **(K–M)** plotter was employed to evaluate the prognostic value of 1,023 combinations, the top 20 was identified and sorted according to the *p* value of **(K-M)**. **(D)** Kaplan-Meier curves stratified by the five-gene prognostic signature in the TCGA cohort **(E)** Risk score distribution and survival overview in the TCGA cohort. **(F)** Principal component analysis (PCA) indicated the risk model has high discriminatory accuracy in distinguishing low-risk group from high-risk groups. **(G)** Pie charts showing the Chi-squared test of clinicopathologic factors for risk model in HCC. **(H)** Univariate and multivariate association of the prognostic model and clinicopathological characteristics with overall survival in the TCGA cohort. **(I)** Time‐dependent ROC curves of the signature and clinical stage in the TCGA cohort.

First, the genes from the PPI network that were significantly associated with the OS of HCC patients from the TCGA cohort were selected as candidate prognostic genes via univariate Cox regression (Figure 5A). Subsequently, a random forest was used to estimate the variable importance values of these prognostic genes and rank them. In this way, the top ten important prognostic genes were identified (Figure 5B). From these ten genes we were able to generate 1,023 risk models. Finally, we compared the -log10 P log-rank value of every possible risk model based on the K-M analysis, and the five-gene signature was identified as the best model (Figure 5C). Subsequently, based on the estimated Cox regression weights, we established a prognostic model, whose formula was computed as Risk score = 0.563 ×*LSM10* + 0.527 × *ATXN2* + 0.025× *SNRPB* + 0.167× *PRPF3* + 0.253× *EDC3*. In this risk model, all the coefficients of the five genes were positive. We then calculated the risk score for each patient in the training set and ranked them. The K-M curves indicated that patients in the high-risk group had significantly worse OS than those in the low-risk group (*p* < 0.001, hazard ratio, HR = 2.34) (Figure 5D). The green dots represent survival, the red dots represent dead patients, and the death risk increased from left to right on the *X*-axis. The results showed that the dead patients were predisposed to be included in the high-risk score group (Figure 5E). Furthermore, principal component analysis (PCA) was applied to examine the power of the five-gene signature in discriminating high- and low-risk patients. The results indicated that the risk model made an excellent distinction between high- and low-risk patients in the TCGA cohort (Figure 5F). The Chi-squared test revealed that the high-risk group showed poorer survival status, clinical stage, pathologic grade, and T stage compared to the low-risk group, suggesting that highly malignant HCC is associated with high risk (Figure 5G). Pathological stages in the prognostic model showed the prognostic value of the gene signature in the TCGA cohort *via* univariate and multivariate Cox regression analyses (Figure 5H). Further, the prognostic value of the five-gene signature was assessed by calculating the area under the curve (AUC) of a time-dependent ROC curve (Figure 5I). The higher the AUC, the better the model’s performance; in the training cohort, the five-gene signature exhibited better predictive efficacy than clinical stage.

**FIGURE 6:**
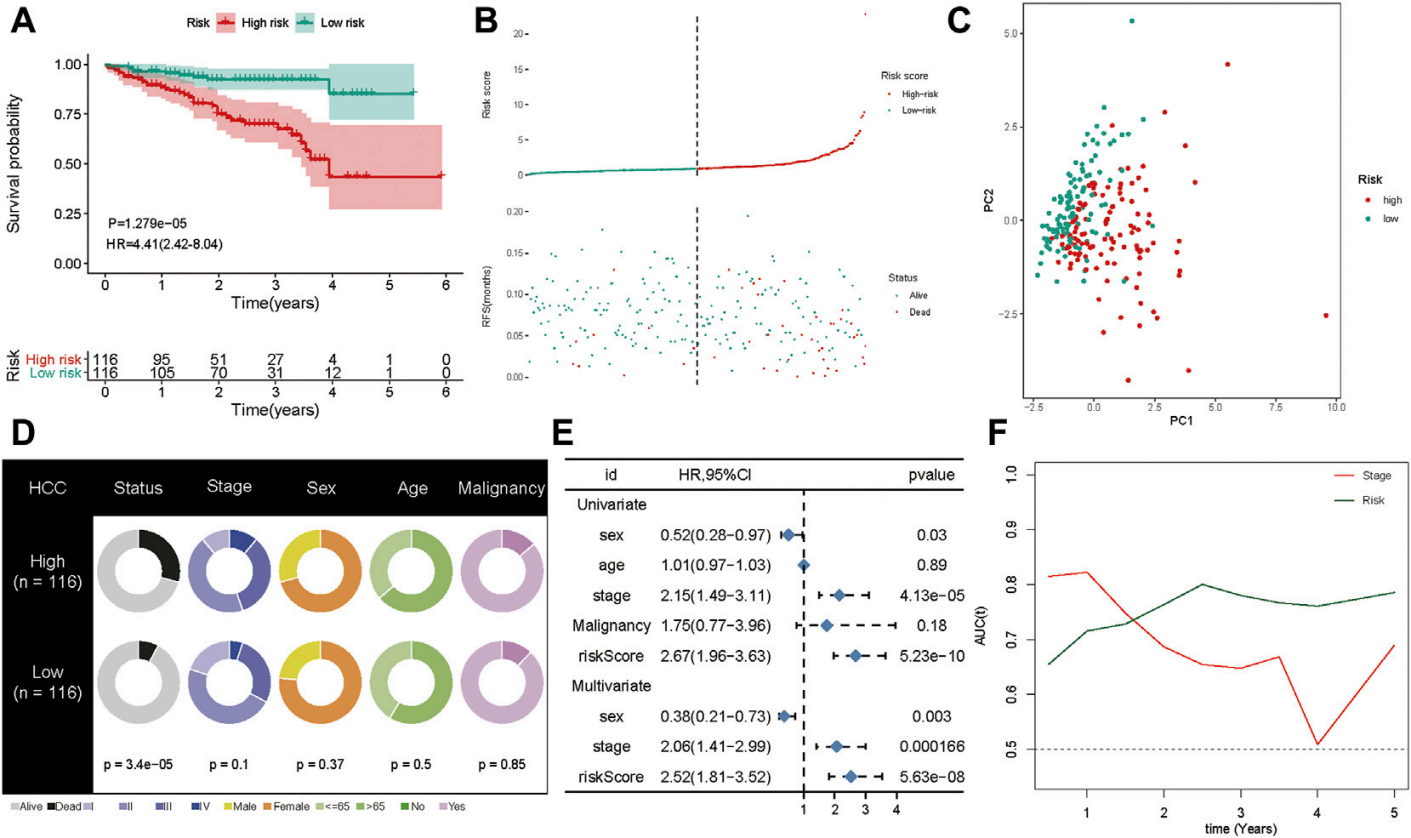
Validation of the gene signature in ICGC cohort. **(A)** The Kaplan-Meier analysis of the signature in ICGC cohort. **(B)** Risk score distribution and survival overview in the ICGC cohort. **(C)** Principal component analysis showed that the high- and low-risk groups exhibited distinct layout modes. **(D)** Pie charts showing the Chi-squared test of clinicopathologic factors for risk model in HCC. **(E)** Univariate and multivariate association of the prognostic model and clinicopathological characteristics with overall survival in the ICGC cohort. **(F)** Time‐dependent ROC curves of the signature and clinical stage in the ICGC cohort.

To confirm the robustness of the five-gene signature, we verified the risk signature in the ICGC cohort. The K-M plots demonstrated that HCC patients with high risk had poorer OS compared to low-risk patients in the validation set (Figure 6A). As seen in the training set, high-risk patients had a higher risk score distribution (Figure 6B). Further, most of the deaths in the validation group were clustered in the high-risk group (Figure 6B). Moreover, PCA showed good discrimination between the high- and low-risk groups (Figure 6C). In addition, the increased value of the risk model significantly correlated with poorer survival status, but not stage classification (Figure 6D). We then performed univariate and multivariate Cox analyses to identify the factors that might affect prognosis. Analysis from the ICGC cohort showed that tumor stage classification, sex, and risk signature were independent factors associated with prognosis (Figure 6E). The time-dependent AUC suggested that the five-gene signature showed favorable performance and stability in predicting the OS of HCC patients (Figure 6F).

### Functional Characteristics of the Five-Gene Signature

**FIGURE 7:**
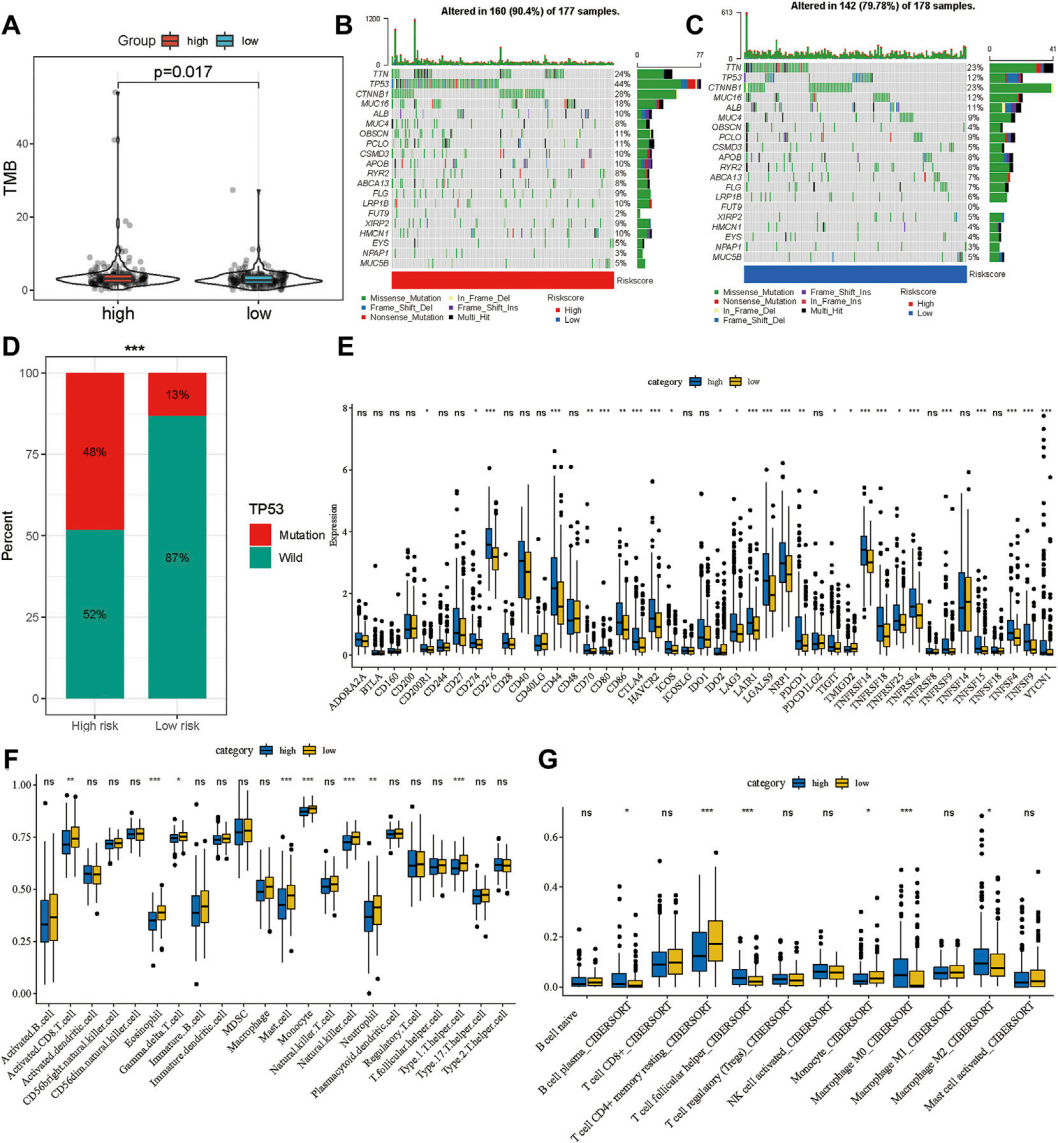
Potential therapeutic value of the gene signature. **(A)** The distribution of tumor mutation burden in the high- and low-risk groups. Waterfall plot of tumor somatic mutations in HCC patients with high-risk scores **(B)** and low-risk scores **(C)**. **(D)** The distribution of high- and low-risk group in mutation and wild TP53. **(E)** The expression landscape of immune checkpoint inhibitor in high- and low-risk group. The distribution of immune cells calculated by ssGSEA **(F)** and CIBERSORT **(G)** in the TCGA cohort. *p < 0.05, **p < 0.01, ***p < 0.001.

Multiple studies have shown that TMB is an emerging biomarker for predicting the efficacy of immunotherapy (Shrestha et al., 2018; Wong et al., 2021). By determining the mutation landscape of the TCGA-LIHC cohort, we found that the high-risk group had a higher TMB than the low-risk group (Figure 7A). Subsequently, we analyzed the distribution of variation of somatic mutations in the high- and low-risk groups in the TCGA cohort. In the analysis, 90.4% of the 177 samples in the high-risk group had a missense mutation, while 79.78% of the 178 samples in the low-risk group had a missense mutation (Figures 7B,C). Further, there was a significant difference in the abundance of *TP53* mutations between the high-risk and the low-risk groups: 44% of patients had *TP53* mutation in the high-risk group, and only 12% had the mutation in the low-risk group. Moreover, the distribution of risk was significantly different between the *TP53* wild-type and *TP53*-mutation groups (Figure 7D). A total of 43 immune checkpoint inhibitors were obtained from published studies, and we compared their expression levels between the high-and low-risk groups, which indicated that most of them were more highly expressed in the high-risk group than in the low-risk group (Figure 7E). Furthermore, the ssGSEA and CIBERSORT methods were applied to delineate the landscape of immune cell infiltrates in HCC patients from the TCGA cohort (Figures 7F,G). The results demonstrated that CD8^+^ T cells, mast cells, monocytes, natural killer cells, and T helper cells had high infiltration in the low-risk group whereas M2 macrophages had high infiltration in the high-risk group (Figures 7F,G). These results explain why the low-risk group had better OS compared to the high-risk group, which might be associated with the relatively low TMB and high immune cell infiltration.

### Construction and Validation of a Predictive Nomogram

**FIGURE 8:**
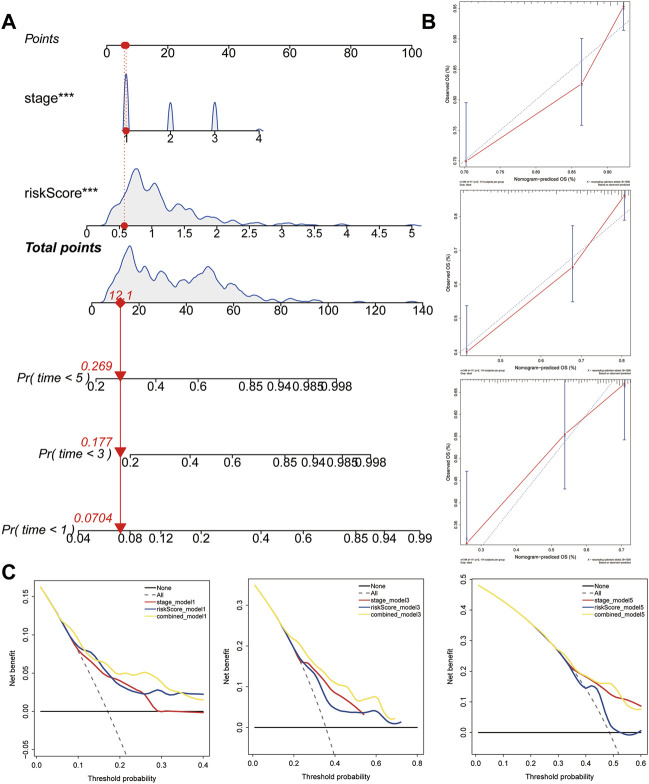
Construction and validation of the predictive nomogram. **(A)** Nomogram predicting 1‐, 3‐ and 5‐years OS for HCC patients. The nomogram is applied by adding up the points identified on the points scale for each variable. The total points projected on the bottom scales indicate the probability of 1‐, 3‐ and 5‐years OS. **(B)** The calibration curve for predicting 1‐, 3‐ and 5‐years OS for patients with HCC. **(C)** Relations between net benefit and threshold probability at 1-year, 3-years, and 5-years survival predictions.

To facilitate the application of the five-gene signature in clinical practice and to predict the probability of 1-, 3-, and 5- year OS, we generated a nomogram composed of two independent prognostic factors (pathologic stage and the five-gene signature) (Figure 8A), based on the multiple regression analyses. The calibration plots were visualized to evaluate the performance of the nomogram in predicting OS. As shown in Figure 8B, the calibration curves were close to the standard curves, indicating that the nomogram performed well in predicting OS. Finally, DCA was used to compare the different prediction models. Graphically, the DCA shows the clinical usefulness of each model based on a continuum of potential thresholds for death (*X*-axis) and the net benefit of using the model to risk-stratify patients (*Y*-axis), while assuming that no patient will die (Figure 8C). In this analysis, the combined model provided a larger net benefit for 1-, 3-, and 5-years OS compared with both the signature and pathological stage models.

### The RNA Expression of *ATXN2, EDC3, LSM10, PRPF3*, and *SNRPB*

**FIGURE 9:**
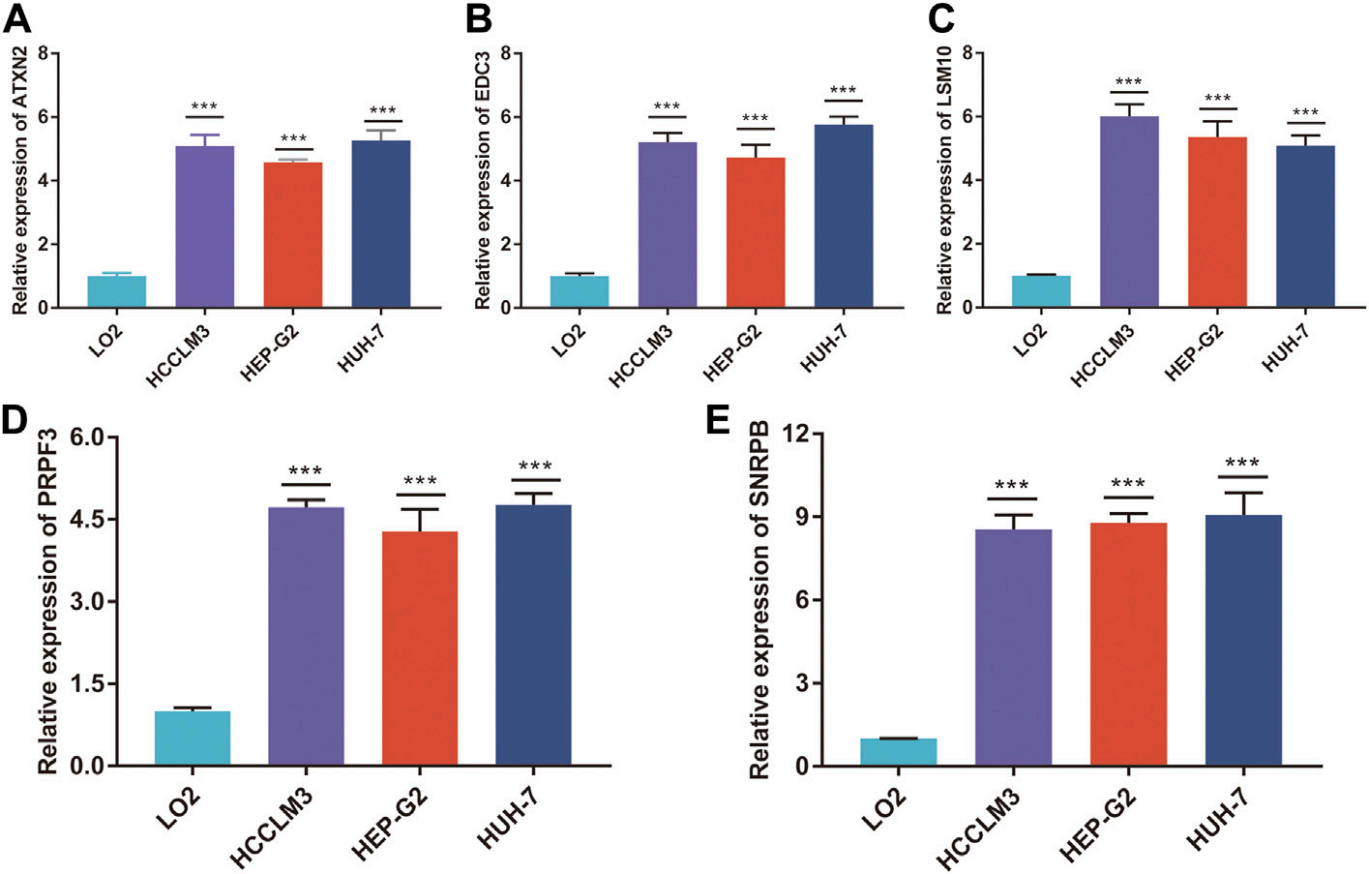
The mRNA expression of five genes. The relative expression of ATXN2 **(A)**, EDC3 **(B)**, LSM10 **(C)**, PRPF3 **(D)**, and SNRPB **(E)** was detected in HCC cell lines and human normal liver cell. ***p < 0.001.

To validate the RNA-seq data, RT-qPCR was used to detect the RNA expression of five genes *in vitro*. These results suggested that *ATXN2, EDC3, LSM10, PRPF3*, and *SNRPB* were upregulated in HCC cell lines, including HCCLM3, Hep-G2, and Huh7, compared to human normal live cell LO2 (Figures 9A–E).

## Discussion

HCC is one of the most fatal solid tumors worldwide (Bray et al., 2018). Although surgery is the first option for HCC patients who can undergo resections, it is still a challenge to deal with recurrence or metastasis, which may shorten the OS by decades (Forner et al., 2018). However, new technologies such as high-throughput genetic sequencing and gene chips give us a more microscopic perspective of complex molecular networks underlying HCC. Further, bioinformatics, which is an emerging approach based on genomics and statistics, can be used to find new predictive information (Berhane et al., 2016; Tai et al., 2019). Wei et al. recently reported a nine-HCV related gene signature for predicting the overall survival of HCC, based on Gene Expression Omnibus (GEO) and TCGA databases (Wei et al., 2021). Liu et al. explored the prognosis value of the mRNA expression-based stemness index (mRNAsi) in HCC, and constructed a new three-gene signature with mRNAsi, based on TCGA and ICGC databases (Liu et al., 2021). In this study, we developed a novel five-gene signature model to predict the outcome of HCC patients based on the spliceosome-related gene expression. Here, the precent study provides a new dimension to understand the malignant process of HCC.

The spliceosome is mainly involved in removing pre-mRNA introns and generating multiple mature mRNA isoforms (Shi, 2017; Wan et al., 2019). Most spliceosome snRNPs contain a common set of core Sm proteins such as *SNRPD1, SNRPD2, SNRPD3, SNRPB, SNRPE, SNRPF,* and *SNRPG* (Papasaikas and Valcárcel, 2016). The Lsm1-7/Pat1 RNA-binding protein complex is an Lsm/Sm protein involved in mRNA decay and acts as a chaperone for spliceosomal RNA (Montemayor et al., 2020). *SNRPB* is an oncogenic splicing factor that participates in the formation of the spliceosome complex SmB/B (Li et al., 2019). Among BRCA1 carriers, the minor allele of rs6138178 in *SNRPB* was significantly associated with lower risk of breast cancer risk (Wang et al., 2010). In prostate cancer, *SNRPB* was initially regarded as a metastasis suppressor gene, as its mRNA expression significantly decreased in metastasizing tumors compared to that in non-tumor tissue (Yi et al., 2009). *SNRPB* was also found to be one of the hubs splicing factors that participate in splicing regulation and are detrimental to the prognosis of glioblastoma, as indicated by a significant correlation between *SNRPB* expression and the alternative terminator of *KIF4A* exon32 (coefficient = 0.70) (Li et al., 2019). Recent studies suggest that *LSM10* may be implicated in tumor-associated modifications in molecular pathways that control histone gene expression during the cell cycle (Ghule et al., 2009). *ATXN2* has been reported to be overexpressed in pancreatic adenocarcinoma (PAAD) tumor tissues, and overexpression of *ATXN2* promotes PADD cell proliferation, migration, and invasion (Fang et al., 2021). *EDC3* plays an important role in regulating RNA decapping and destruction in cancer progression, and it can also promote tumor growth and invasion (Bearss et al., 2021). However, the expression level and clinical value of *LSM10, ATXN2*, and *EDC3* in HCC have not been explored. Our results showed that these genes were highly expressed in HCC tumor tissue, and high expression was associated with poor OS. Therefore, we infer that *LSM10, ATXN2*, and *EDC3* may act as upstream molecules to promote HCC progression. It has been found that *PRPF3* expression was increased in HCC tissues, and could regulate *HNF4*alpha expression and reinforce the proliferative response to epidermal growth factors (Niehof and Borlak, 2008; Liu et al., 2020a).

Although immunotherapy has been recently used as cancer treatment in clinical practice, studies have found that only approximately 20% of patients with solid tumors may benefit from this treatment (Zongyi and Xiaowu, 2020). TMB, immune cell infiltration, and immune checkpoint inhibitors play critical roles in anti-tumor immune responses and immunotherapy (Zheng et al., 2017; Wu et al., 2019; Liu et al., 2020b; Zhang et al., 2020). Several studies have shown evaluation of TMB, CD8^+^ T cells, CD4^+^ T cells, M2 macrophages, *CD276, CD274*, and *CTLA4* may help in predicting immunotherapy efficacy (Seaman et al., 2017; Ma et al., 2019; Pai et al., 2019; Wu et al., 2021). Recent reports suggest that immune-related gene signatures correlate with the immunophenotype, which can predict the effectiveness of immunotherapy (Cancer Genome Atlas Research Network, 2017; Chen et al., 2020). In this study, we constructed a five spliceosome-related gene risk model, and the high-risk group showed higher TMB and immune checkpoint inhibitor expression and lower immune cell infiltration. This also explains why HCC patients in the high-risk group had worse OS.

In conclusion, we delineated the expression landscape of spliceosome-related genes and revealed the prognostic value of these genes in HCC. We then constructed a spliceosome-related gene signature and developed a nomogram to predict the OS of patients with HCC. We also explored the relationship between TMB, immune checkpoint inhibitors, immune cells, and risk score. Then the five genes were overexpressed in HCC cells than normal liver cell.

## Data Availability

The original contributions presented in the study are included in the article/Supplementary Material, further inquiries can be directed to the corresponding author.
