# Supplementary material for: Determining the Prognostic Value of Spliceosome-Related Genes in Hepatocellular Carcinoma Patients
Source: Front Mol Biosci. 2022 Feb 24;9:759792. doi: 10.3389/fmolb.2022.759792 (PMC8907852; doi:10.3389/fmolb.2022.759792)
Supplement: Supplementary file 1 [file Table2.DOCX]

| Gene | Sequence |
| --- | --- |
| ATXN2 F | CTCTACTATGCCTAAACGC |
| ATXN2 R | GACTGGTCCTTGCTACTG |
| EDC3 F | AGGGCAGGTGACATTACG |
| EDC3 R | TCTTAGGGATATTCTGAGGG |
| LSM10 F | CCCACGGACGCATAGACAA |
| LSM10 R | AGTTTCGCACCCGATGGA |
| PRPF3 F | AAGAGGGTCCTGGGTTT |
| PRPF3 R | CTGCTCCTGTCACTGCT |
| SNRPB F | GATCTTCATTGGCACCTTC |
| SNRPB R | TCTTCCCTTTCTGCTTGTT |
| β-actin F | GGCACCCAGCACAATGAA |
| β-actin R | TAGAAGCATTTGCGGTGG |

Table S1. The primer sequence of genes.
